# The binding of cellulase variants to dislocations: a semi-quantitative analysis based on CLSM (confocal laser scanning microscopy) images

**DOI:** 10.1186/s13568-015-0165-9

**Published:** 2015-12-01

**Authors:** Budi J. Hidayat, Carmen Weisskopf, Claus Felby, Katja S. Johansen, Lisbeth G. Thygesen

**Affiliations:** UniBio a/s, Billedskaerervej 8, 5230 Odense M, Denmark; Department of Biomaterials, Max Planck Institute of Colloids and Interfaces, Am Műhlenberg 1, Potsdam, 14476 Germany; Department of Geosciences and Natural Resource Management, University of Copenhagen, Rolighedsvej 23, 1958 Frederiksberg, Denmark; Division of Industrial Biotechnology, Chalmers University of Technology, Kemivägen 10, 41296 Göteborg, Sweden

**Keywords:** Cellulase binding, Fluorescence-labelled enzymes, Dislocations, Confocal laser scanning microscopy, Ratio imaging, Semi-quantitative analysis

## Abstract

Binding of enzymes to the substrate is the first step in enzymatic hydrolysis of lignocellulose, a key process within biorefining. During this process elongated plant cells such as fibers and tracheids have been found to break into segments at irregular cell wall regions known as dislocations or slip planes. Here we study whether cellulases bind to dislocations to a higher extent than to the surrounding cell wall. The binding of fluorescently labelled cellobiohydrolases and endoglucanases to filter paper fibers was investigated using confocal laser scanning microscopy and a ratiometric method was developed to assess and quantify the abundance of the binding of cellulases to dislocations as compared to the surrounding cell wall. Only *Humicola insolens* EGV was found to have stronger binding preference to dislocations than to the surrounding cell wall, while no difference in binding affinity was seen for any of the other cellulose variants included in the study (*H. insolens* EGV variants, *Trichoderma reesei* CBHI, CBHII and EGII). This result favours the hypothesis that fibers break at dislocations during the initial phase of hydrolysis mostly due to mechanical failure rather than as a result of faster degradation at these locations.

## Introduction

Dislocations or slip planes are irregular regions within in the wall of thick-walled plant cells such as fibers and tracheids. Within cellulosic bioethanol production, dislocations have been found to be important for efficient particle size reduction during the initial phase of the enzymatic hydrolysis process, as they are weak regions where fibers break. In this study we look into whether the binding affinities of different cellulase variants vary between dislocations and the surrounding cell wall.

Albeit dislocations have been known to science for more than 100 years (Höhnel 1884), their exact structure, formation and role in the living plant remain only partially known. What has been found so far is that dislocations are present in the load bearing structures of living plants, i.e. stems and branches, but that they may also be introduced during post-harvest plant biomass processing, especially as a result of compression stress (Nyholm et al. 2001; Ander et al. 2005; Terziev et al. 2005; Thygesen and Asgharipour 2008; Thygesen 2011). Regarding the chemical composition of the cell wall, dislocations do not seem to differ from the surrounding material, but the cellulose microfibril angle is different within dislocations (Thygesen and Gierlinger 2013). Dislocations have been found to be birefringent just like the surrounding cell wall (Thygesen et al. 2011), so ordered cellulose structures must be present although a well-established increased susceptibility towards acid and enzymatic hydrolysis (Ander 2002; Ander et al. 2005, 2008; Frölander et al. 1969; Thygesen 2008; Thygesen et al. 2011; Wallace 2006) has been interpreted as indicative of a more amorphous cellulose structure (Ander 2002; Cochaux and d’Aveni 1995). One can speculate that compression stress applied in the longitudinal direction of the plant cell gives rise to buckling of the cell wall, but that the cellulose remains piece-wise ordered in segments that follow the buckling. Cracks have been found to be present in and around severe dislocations (Terziev et al. 2005), which could affect how accessible they are to chemicals and enzymes and perhaps help explain the increased susceptibility to hydrolysis. Dislocations have been suggested to be present in both the S1 and S2 layers of the plant cell wall (Hartler 1995; Ander et al. 2008). Comparison of scanning electron microscopy and polarized light microscopy results has shown that only larger dislocations are visible on the surface of native fibers (Thygesen et al. 2006).

In order for biorefining schemes involving the production of cellulosic ethanol to be economically viable, it is necessary to run the enzymatic hydrolysis step at high dry matter contents in order to decrease the downstream cost of isolating the products. The input material is consequently more like a paste than a slurry, but needs to be liquefied in order to become pumpable. This can be achieved within hours if enzymatic hydrolysis is combined with the right type of mixing (Jørgensen et al. 2007). It is known that liquefaction is linked to particle size reduction, and that segmentation of the long, thick-walled plant cells at dislocations is important in this (Frölander et al. 1969; Thygesen et al. 2011). For example, Skovgaard et al. (2014) found that for saccharification of hydrothermally pretreated wheat straw, viscosity and mean fiber length both decreased dramatically during the first hour of hydrolysis.

Industrial scale saccharification at high dry matter level involves simultaneous enzymatic hydrolysis and mechanical agitation of the slurry. The question we are interested in studying is whether plant fibers break at dislocations during this process due to a preferred binding of enzymes and subsequent higher rate of hydrolysis at these locations, or whether it is mostly a mechanical mechanism with fibers simply breaking at weak points.

Enzymatic hydrolysis at dislocations has been inferred from (1) visual observation of labelled enzymes binding to dislocations, (2) monitoring the hydrolysis of a single fiber using microscopy, or (3) analysing the physical dimension of fibers during or after hydrolysis (Hidayat et al. 2012). Here we pursue the first, most direct approach and develop a semi-quantitative CLSM (confocal laser scanning microscopy)-based method for measuring the binding of fluorescently labelled cellulases to dislocations compared to the surrounding cell wall.

The increasing use of fluorescence microscopy for the study of enzyme binding to lignocellulosic materials was reviewed by Moran-Mirabal (2013). Both fluorescence (e.g. Pinto et al. 2007; Moran-Mirabal et al. 2008) and confocal laser scanning (e.g. Zhu et al. 2011) microscopy have been used to determine the binding characteristics of cellulases or their CBMs (carbohydrate binding domains) to cellulosic or lignocellulosic substrates. The techniques developed by these authors allow the observation of cellulases binding to different structures on the surface or within fibers, as well as a relative determination of binding coefficients for different locations on a fiber. For example, Zhu et al. (2011) found that different cellulose morphologies saturated at different times, suggesting that not all binding sites are equally accessible. The increasing emission intensity over time of the cellulose surface was interpreted as an increasing number of enzyme molecules bound to the fibers. Furthermore, Luterbacher et al. (2015) recently used a CLSM-based technique to determine cellulase activity on different substrates. The use of CLSM differs from traditional binding studies (e.g. Filonova et al. 2007; Hildén et al. 2003) where the amount of bound enzyme is estimated based on what is present in the supernatant after a certain incubation period, giving no information on the location of the bound enzymes.

In this work we measure the adsorption to filter paper fibers of the following fluorescently labelled endo- and exo-cellulases: GH45 endoglucanase from *H. insolens (EGV)*, GH7 endoglucanase from *Trichoderma reesei* (EGII), GH7 *T. reesei* cellobiohydrolase (CBHI) and GH6 *T. reesei* cellobiohydrolase (CBHII). The binding of modified *H. insolens* EGV and *T. reesei* CBHI, i.e. these enyzmes without the cellulose binding module or with an inactive catalytic domain, were also investigated. For each, the binding to dislocations and to neighbouring cell wall regions is compared. The binding of the enzymes was compared after a certain time of incubation (i.e. at a single time point) as binding kinetics was not the focus of this study. Filter paper fibers was chosen as a model substrate having still a relatively intact cellulose structure, while being devoid of lignin and hemicellulose. In the nanometer range, the average pore size within dislocations was found not to be significantly different from that in the surrounding cell wall (Hidayat et al. 2013) for this type of fiber. However, a recent study on flax fibers documented that micrometer-size voids may be present within large dislocations (Zhang et al. 2015).

## Materials and methods

### Enzymes and fluorophore labelling

The enzymes and proteins used in this work were gifts from Novozymes A/S and are listed in Table 1. These enzymes/proteins were glycosylated by the expression host and no effort was done to alter the glycosylation after the expression.

**Table 1:** Enzymes/proteins used in this study

Enzymes	MW (kDa)	Number of constituent amino acids	Number of CLSM images	
			Non washed samples	Washed samples
*Humicola insolens* EGV	43	284	12	8
*Humicola insolens* EGV, non CBM	31.2	213	21	11
*Humicola insolens* EGV, non CBM, inactive^a^	31.2	213	15	9
Antibody-CBM_EGV_^b^	31.6	216	18	12
*Trichoderma reesei* CBHI	68	497	8	6
*Trichoderma reesei* CBHI, non CBM	50	436	11	3
*Trichoderma reesei* CBHII	58	365	10	4
*Trichoderma reesei* EGII	48	398	11	4

The size of the enzymes and the number of CLSM images obtained for each enzyme are shown

^a^Is a *H. insolens* EGV Active Domain with a single mutation at its active site

^b^Is a chimeric protein having similar size with the Active Domain of *H. insolens* EGV and constructed by combining CBM from *H. insolens* EGV and an antibody molecule

The enzymes were crosslinked with the fluorophore Dylight633 (Thermoscientific, Rockford, Illinois, USA), following the procedure given by the manufacturer. The crosslinked product was purified by a minimum of 8 rounds of centrifugation using microcentrifuge tubes. The enzyme concentration and the degree of fluorophore labelling were determined following the procedure suggested by the manufacturer. A degree of labelling between 0.6 and 1.4 mol fluorophore/mol enzyme was typically obtained.

### Microscopy

When labelled enzyme molecules are spotted onto a dry fiber, they may bind to loose material situated around the fiber and may to some extent hinder others to reach parts of the fiber cell wall. In order to overcome this issue, all fibers were incubated in diluted enzyme solution for 1 h.

Dissected cellulose filter paper (Munktell and Filtrak GmbH, Bärenstein, Germany) was incubated in 20 μl of diluted enzyme (40–100 nM) solution for 1 h in an eppendorf tube. Following this the filter paper was either spotted on a glass slide or washed 2 times with MQ water, each for 5 min, in another eppendorf tube prior to spotting. Occasional tapping was performed during the washing to provide water movement. Twenty microliter MQ-water was added to the glass slide to immerse the fibers before a cover slip was applied. The use of MQ-water, instead of buffer, is deemed justified as in this work we are interested in the binding of the enzymes, not their activity which can be strongly affected by pH.

A Leica SP2 Confocal Laser Scanning Microscope (Leica Microsystems GmbH, Wetzlar, Germany) equipped with a 63× water immersion objective (Numerical Aperture = 1.2) was used for imaging with either 1 or 2 x electronic magnification. A 1024 × 1024 pixel format was applied leading to a pixel size of ca. 232 × 232 or 116 × 116 nm. The pinhole size was kept at 1 Airy Unit for all experiments. Typically a 20 % laser intensity and 722 or 900 V gain was used. Excitation was performed at 633 nm and emission was collected between 650–750 and 644–800 nm. This variation was to compensate for the higher/lower emission from the fluorophores (e.g. due to different degree of labelling). Under all settings, however, no emission was registered when fluorophore-labeled enzyme was not present. This absence of detected autofluorescence removed the need to use a special algorithm to separate autofluorescence from real signal (Moran-Mirabal et al. 2008).

The typical resolution obtained under the CLSM setting is approximately 270 nm laterally and around 740 nm vertically [calculated based on the Abbe formula for lateral resolution and other relevant formula for vertical resolution (Wilhelm 2010)], taking into consideration the numerical aperture of the microscope’s objective (NA = 1.2) and the excitation wavelength of 633 nm. Typically between 8 and 12 optical planes, or slices, were captured for each fiber, making a 3-dimensional representation of the fibers and surrounding area, which we here call an image cube.

**Fig. 1:**
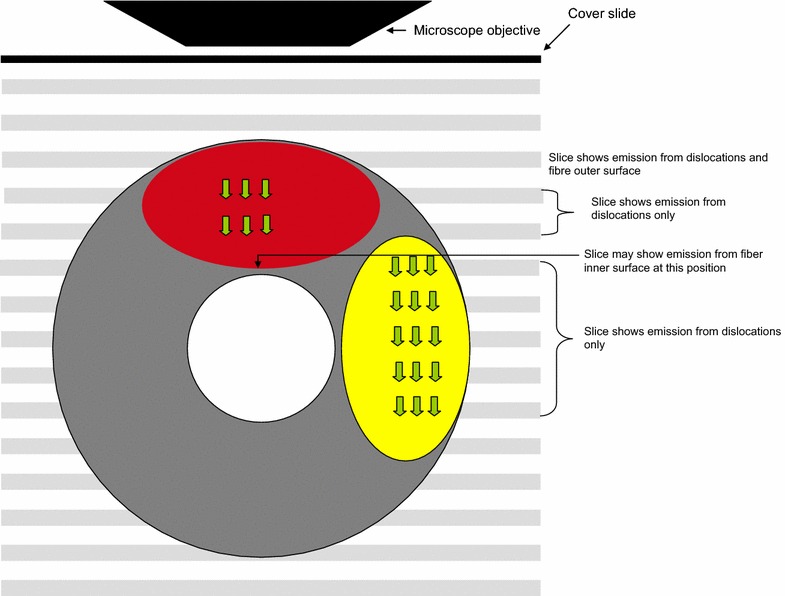
A schematic drawing depicting the cross section of a latewood fibers (Ø = 24 μm, Ø_lumen_ = 8 μm) lying *horizontally* under the microscope and the position of ROIs (Regions of Interest, *green arrows*) showing dislocations. Dislocations (*red*, *yellow areas*) are here shown not to extend across the fibers, although this may also be the case. Selecting the *yellow* dislocation means more slices can be included for calculation. If the *red* dislocation is chosen, only 2 slices can be included due to emission interference from the outer and inner surface of the fiber. *Grey lines* slices of CLSM imaging. *Fifteen slices* are scanned covering a 30 μm depth, giving a 2 μm distance between slices

The locations of dislocations were found using a cross-polarized light image obtained using the built-in transmission detector capturing data from the same position as the CLSM detector. This detector, however, captured a transmission representation of fiber in contrast to the CLSM images which corresponded to a certain confocal plane. Only fibers with large dislocations located at the side of the fibers (when seen from above through the microscope, as opposed to dislocations present on top of the fibers; see Fig. 1) were used.

### Data processing

Within an image cube (i.e. the whole 3D image obtained by stacking all the optical slices), ROIs (Regions of Interest) were selected for three types of positions: bulk solution, normal cell wall and dislocations. Bulk solution represents area where there is no fiber present, while normal cell wall is loosely defined as any area not showing dislocations under polarized microscopy. A ROI can be understood as an area in the lateral x–y plane. For each type of position three ROIs were selected. In the axial (z-) direction, a number of slices of interest (SOIs) were selected for each ROI. The concepts of ROIs and SOIs are illustrated in Fig. 1. SOIs were selected manually to exclude slices in the axial (z-) direction showing fiber surface. Fiber surface typically exhibits more intense fluorescence. The extra fluorescence must have originated from the binding of enzymes on the surface and fines protruding out of the surface and may confound fluorescence originating from specific binding to dislocations close to the surface. For this reason we selected only.

**Fig. 2:**
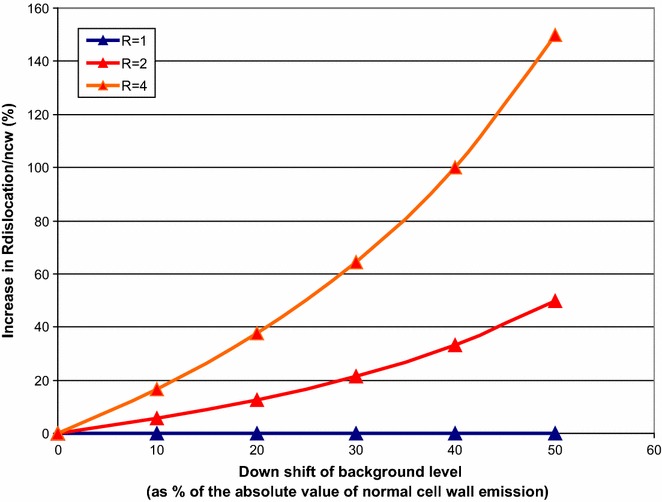
Sensitivity of R values to changes in the absolute zero of the background level. Higher R values are more prone to shift in the absolute zero of the background level

slices that contained lumina. This principle minimizes the possibility of selecting a ROI positioned too close to the fiber’s outer surface in the lateral x–y plane, having in mind that (1) the round form of a fiber means that the width of the fiber at some slices (toward the top and bottom of the z-axis) would be less than that indicated by the transmission detector image and (2) in slices where lumen appears, the fiber is close to or at its widest. This also guarantees that a large part, if not all, of the signal collected comes from the S2 wall.

Dislocations are assumed to extend at least throughout half the cross section of a fiber, which is a reasonable assumption since large dislocations are known to extend all the way across a fiber (Forgacs 1961; Nyholm et al. 2001). Typically between 4 and 6 SOIs were selected per image cube. The total thickness represented by these SOIs may vary. The use of a set of SOIs instead of only a single optical slice includes a greater part of the dislocation and consequently reduces the representation error.

ROI selection, as described above, was performed manually using the software LeicaLite (Leica Microsystems GmbH, Wetzlar, Germany) and the data was transferred to Excel (Microsoft, Redmond, Washington, USA) for further processing.

The binding to dislocations compared to the normal cell wall in a SOI is described by the ratio between average emission values from within dislocations (as collected from three ROIs) and average emission values from normal cell wall (as collected from three ROIs), termed R_dislocations/normal cell wall_.

R_dislocations/normal cell wall_ from all SOIs were then averaged to obtain a single R reflecting the whole set of SOIs. This way of calculating R was chosen against the alternative sequence of first averaging all the emissions over the whole set of SOIs before rationing and then averaging the values obtained from 3 ROIs. This latter procedure assumes no differential modulation of emission intensity due to absorption and scattering across the cell wall, an assumption we could not make. The standard deviation of the R values thus reflects not only the inherent heterogeneity within a fiber and the measurement error, but also the result of error propagation incurred during the multistep calculation described above. In order to compensate for these, we inspected a relatively large number of fibers for each enzyme/protein and treatment combination, resulting in a number of R values (see Table 1). We treat these R values as individual datapoints (instead of an average having standard deviation) to enable us to focus on the spread of R values between fibers subjected to the same treatment.

Normally it is not possible to compare two fluorescence intensity values taken from different CLSM images, unless rigid procedures are applied to remove differences in levels of background emission, degrees of enzyme labelling, thickness of CLSM slices or other differences in CLSM settings. However, by comparing one fluorescence emission to another in the same SOI, such as done in this work for fluorescence emission from dislocations and from surrounding cell wall (as R), it is possible to extract information about the relative binding affinity of a fluorescently labelled enzyme. This relative binding affinity can then be compared with R values extracted from other images. That the enzyme binding to surrounding cell walls exhibits different “absolute” fluorescence intensities in different images (i.e. different fibers) is irrelevant in this particular study, as what is sought is whether one or more enzymes (or protein) has increased binding affinity to dislocations, as compared to the surrounding cell wall. Any variation in the degree of enzyme labelling and in the CLSM slice thickness will affect fluorescence emissions from dislocations and from surrounding cell wall in the same way. As these emissions are the numerator and denominator of the R formula, the difference caused by these error sources will be cancelled out.

The different setting of the zero level of the background value may provide a source of error that affect R. While our setting ensures that autofluorescence is not observed throughout this study, it is possible that small variations of the zero baseline occurs e.g. due to occasional instrument adjustments. In Fig. 2 the relation between the change in background absolute zero level and the increase in R is depicted. It can be seen that a ~30 % down shift in the position of absolute background value can be accepted before the disparity between the resulting R_with downshift_ and R_no downshift_ values exceeds 21 % for R = 2. The figure also shows that if R is close to 1 then the change in R is negligible. As the R values we obtained (see “Results”) are not higher than 2, we conclude that this type of error did not affect the interpretation of our data.

## Results

**Fig. 3:**
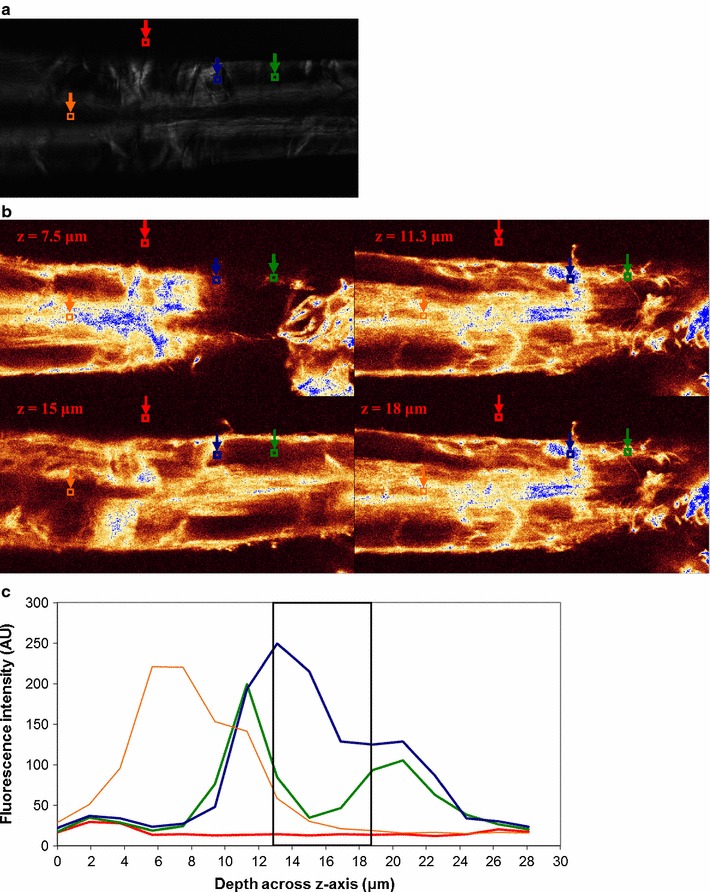
*Humicola insolens* EGV binding to a fiber. **a** PLM image showing dislocations; ROIs are shown for bulk solution (*red square*), normal cell wall (*green square*), dislocations (*blue square*), and lumen (*orange square*). **b** CLSM images showing binding at 4 different levels across *z-axis* of the microscope as shown in **c**. **c** Binding profile across *z-axis* within ROIs shown in **a** (same colour-code). SOIs (Slices of Interest; 4 slices) between 13.1 and 18.8 μm (*boxed region*) were selected for R calculation. Fluorescence intensity level in arbitrary unit. *H. insolens* EGV shows a strong binding preference for dislocations compared to the surrounding normal cell wall. The size of the images is 119.16 × 59.58 μm^2^

**Fig. 4:**
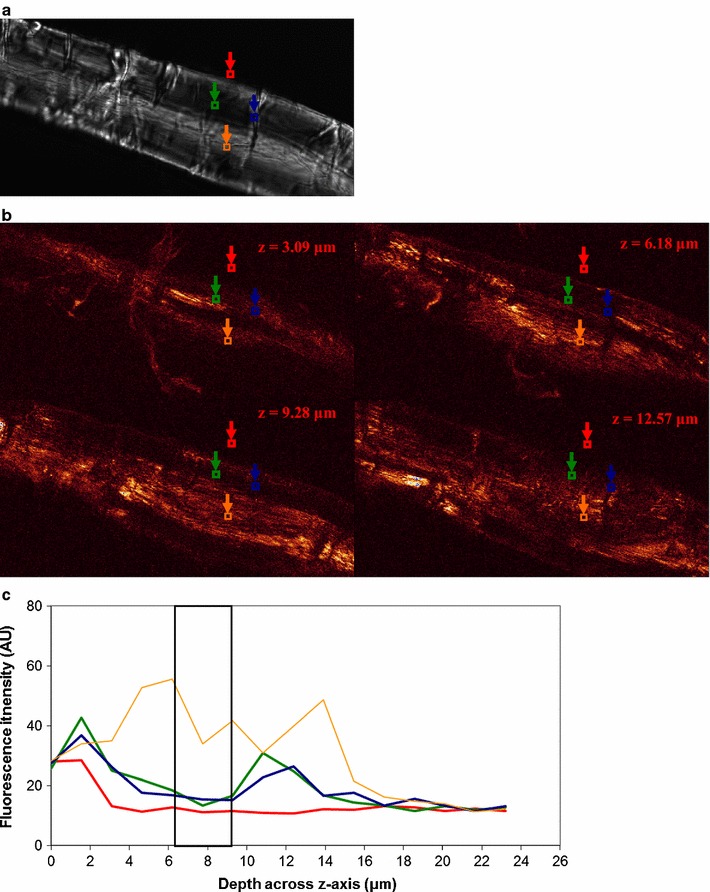
*Humicola insolens* EGV (non CBM) binding to a fiber. **a** PLM image showing dislocations; ROIs are shown for bulk solution (*red square*), normal cell wall (*green square*), dislocations (*blue square*), and lumen (*orange square*). **b** CLSM images showing binding at 4 different levels across *z-axis* of the microscope as shown in **c**. **c** Binding profile across *z-axis* within ROIs shown in **a** (same colour-code). SOIs (Slices of Interest; 4 slices) between 6.2–9.3 μm (*boxed region*) were selected for R calculation. Fluorescence intensity level in arbitrary unit. *H. insolens* (non CBM) does not show increased binding to dislocations. The size of the images is 119.16 × 59.58 μm^2^

**Fig. 5:**
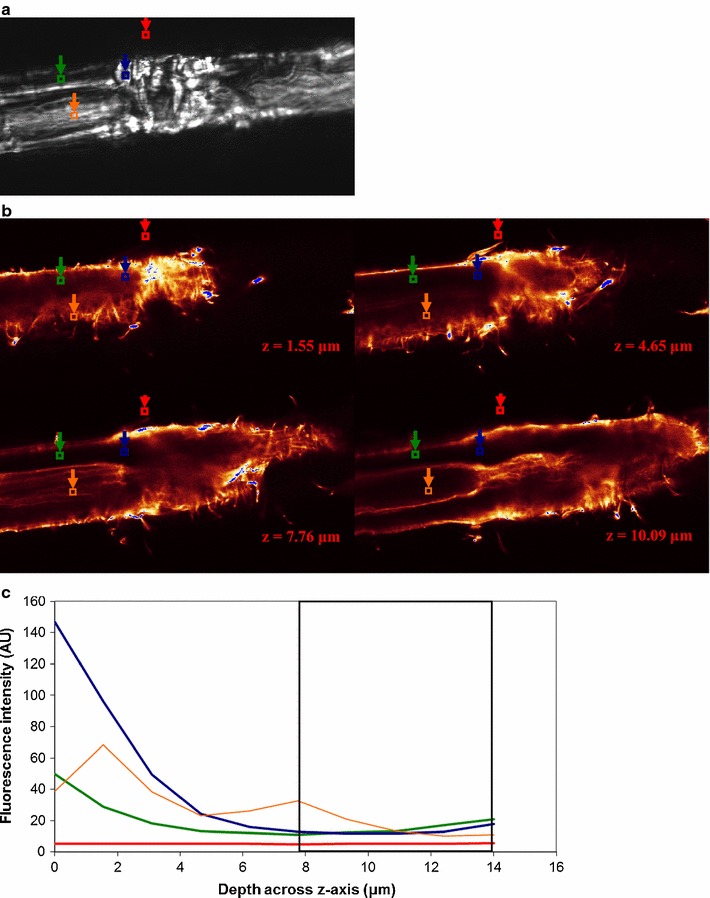
*Trichoderma reesei* CBHI binding to a fiber. **a** PLM image showing dislocations; ROIs are shown for bulk solution (*red square*), normal cell wall (*green square*), dislocations (*blue square*), and lumen (*orange square*). **b** CLSM images showing binding at 4 different levels across *z-axis* of the microscope as shown in **c**. **c** Binding profile across *z-axis* within ROIs shown in **a** (same colour-code). SOIs (Slices of Interest; 5 slices) between 7.8 and 14 μm (*boxed region*) were selected for R calculation. Fluorescence intensity level in arbitrary unit. *T. reesei* CBHI does not show increased binding to dislocations. The size of the images is 119.16 × 59.58 μm^2^

**Fig. 6:**
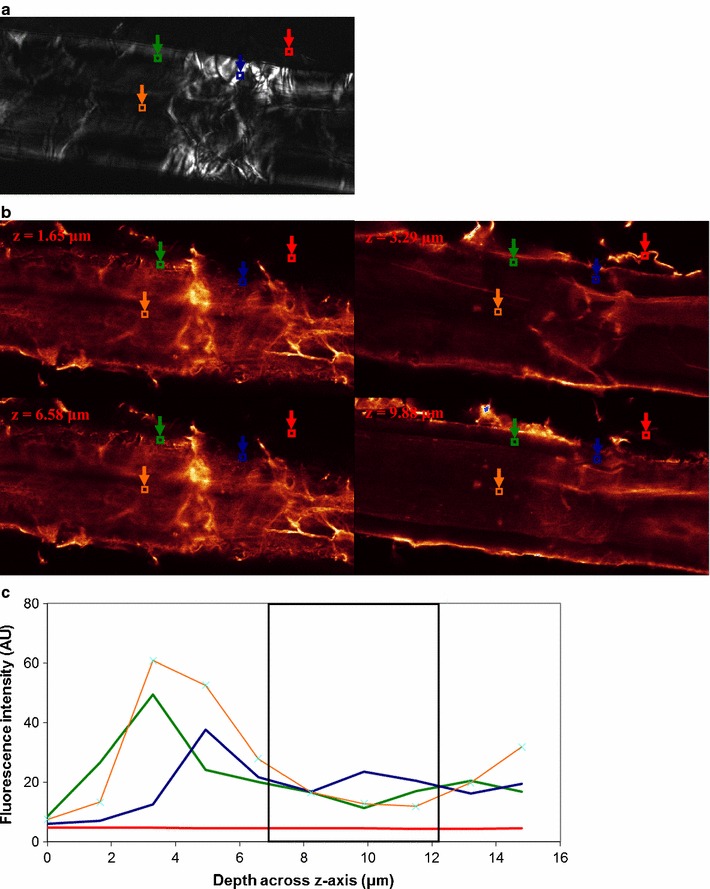
*Trichoderma reesei* CBHI (non CBM) binding to a fiber. **a** PLM image showing dislocations; ROIs are shown for bulk solution (*red square*), normal cell wall (*green square*), dislocations (*blue square*), and lumen (*orange square*). **b** CLSM images showing binding at 4 different levels across *z-axis* of the microscope as shown in **c**. **c** Binding profile across z-axis within ROIs shown in **a** (same colour-code). SOI (Slices of Interest; 4 slices) between 6.5 and 11.5 μm (*boxed region*) were selected for R calculation. Fluorescence intensity level in arbitrary unit. *T. reesei* CBHI (non CBM) does not show increased binding to the dislocations. The size of the images is 119.16 × 59.58 μm^2^

Enzyme binding to fibers is shown for *H. insolens* EGV (Fig. 3), *H. insolens* EGV without CBM (Fig. 4), *T. reesei* CBHI (Fig. 5) and *T. reesei* CBHI without CBM (Fig. 6). To illustrate the method outlined in the “Materials and methods” section, a single ROI (instead of the actual three ROIs) for each of the three types of positions is depicted, and the fluorescence profile, representing enzyme binding, over the z-axis of the fiber within the ROI is shown (Figs. 3c, 4c, 5c, 6c); to keep consistency with the fluorescence level in their respective CLSM images, the fluorescence level shown in the y-axis of these figures is not normalized. SOIs were selected based on these profiles, having in mind the consideration discussed in the Microscopy part of the Materials and methods section. It appears that for all enzymes included in the study most of the fluorescence emission (and thereby the enzyme) is localized within ca. 1 μm of the fiber surface (see Figs. 3b, 4b, 5b, 6b). The level of emission as seen on the fiber surfaces is not observed within the fibers, except in some cases within dislocations. However, the inner part of the normal cell wall shows higher emission intensity than the bulk solution of washed samples, suggesting that some enzymes found their way inside, as also observed by Pinto et al. (2007, 2008). Enzymes bound to fines protruding off fibers are also seen, again concurring with the work of Pinto et al. (2008).

**Fig. 7:**
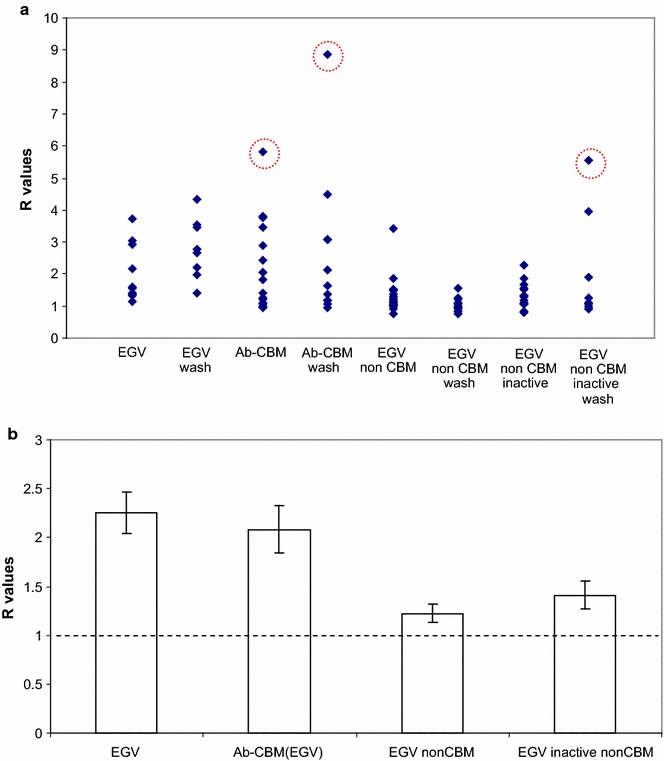
**a** Plot of R values of *H. insolens* EGV-related enzymes/proteins. *Circled data points* indicate suspected outliers, **b** *Bar graph* made of data points in **a** after omission of suspected outliers and pooling of the unwashed and washed samples. Standard error of the mean is used to describe data variation. Enzymes/protein containing CBM from *H. insolens* EGV (EGV) show higher R (around 2) compared to those that do not have this binding module

**Fig. 8:**
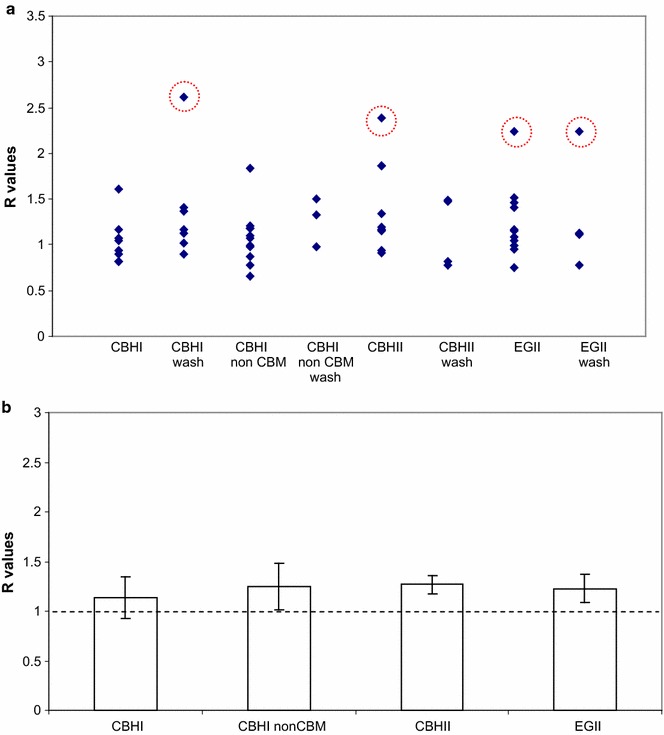
**a** Plot of R values of *T. reesei* CBHI, CBHI without CBM, CBHII and EGII. Note that maximum *y-axis* scale is different than in the equivalent graph in Fig. 7. *Circled data points* indicate suspected outliers, **b** *Bar graph* made of data points in **a** after omission of suspected outliers and pooling of the unwashed and washed samples. Standard error of the mean is used to describe data variation. Essentially the values are around 1.1–1.2 (see text) with none reaching R = 2

R values obtained from each measurement are shown as datapoints in Figs. 7a and 8a. Mean R values are calculated for each enzyme/protein and treatment combination after exclusion of what we believe are outliers (circled data in Figs. 7a and 8a). As no difference was found between the washed and unwashed samples subjected to the same enzyme/protein (*t-*test, 95 % confidence interval, not shown) these data are pooled and the mean for each enzyme/protein was calculated and is shown as bars in Figs. 7b and 8b. As we are interested in the precision of the estimates for the means, we choose to show SEM (standard error of measurement, standard deviation/n^½^, where n = number of samples) instead of the standard deviation, which characterizes the variability of the data. By showing SEM we also account for the different number of samples associated with each enzyme/protein.

## Discussion

Figures 7b and 8b show that while R values for *H. insolens* EGV and Antibody-CBM_EGV_ reach up to ca. 2, none of the R values of exocellulases or the other endoglucanase tested in this work reach this level. The difference between R values of *H. insolens* EGV and Antibody-CBM_EGV_ in one hand, and the rest of the enzymes on the other hand is significant. The t-test (95 % confidence interval) to determine the significance of the difference between the R of Antibody-CBM_EGV_ and the Rs of the two non-CBM EGVs is given as an example in Table 2. Thus *H. insolens* EGV and Antibody-CBM_EGV_ clearly show preferred binding to dislocations. As the non-CBM *H. insolens* EGV shows lower R values than those having CBM of *H. insolens* EGV, we are compelled to conclude that this CBM plays a role in the increased binding to dislocations.

**Table 2:** t-test to determine the significance of the difference between the mean of R values of antibody-CBM_EGV_ and non-CBM EGVs

	Antibody-CBM_EGV_	EGV without CBM	EGV inactive without CBM	Antibody-CBM_EGV_ vs EGV without CBM	Antibody-CBM_EGV_ vs EGV inactive without CBM
Mean R	2.08	1.22	1.41		
Number of samples	28	28	22		
Total samples				28 + 28 = 56	28 + 22 = 50
2-Tailed probability points of t-distribution at 5 % level				2.01	2.01
Standard error of measurement*				0.26	0.28
Mean difference				2.08 − 1.22 = 0.86	2.08 − 1.41 = 0.67
95 % confidence interval for the difference between population means				0.86 ± 2.01 × 0.26 or 0.43–1.26	0.67 ± 2.01 × 0.28 or 0.19–1.15
				As the 95 % confidence interval does not include 0, the difference between the two means is real	

*Calculated according to the formula SEM = (s^2^ × (1/n1 + 1/n2))^0.5, where s is the combined variance of the two population involved = (SS1 + SS2)/(n1 + n2 − 2), while SS1 and SS2 are the sum of squares of the respective population and n1 and n2 are the number of samples of the respective population

CBM of *H. insolens* EGV is a member of the CBM1 family, a family which is shared by all the cellulases tested in this investigation (and by almost all fungal cellulases). That these other cellulases do not show similar preference to dislocations cannot be readily explained. Based on a BLAST search for protein homology (Altschul et al. 1990), the CBM1 of *H. insolens* GH45 endoglucanase shares 55–63 % amino acid residue identity with the other CBM1s of the other cellulases tested in this investigation, while among themselves the other CBMs share 67–77 % amino acid residues. It remains a question if the difference in protein structure, represented by this sequence difference could result in the differences observed in binding affinity. Thus, a separate study is required to be able to explain *why* the CBM of *H. insolens* EGV has higher binding specificity than that of other enzymes tested in the present study.

One can speculate that dislocations exhibit a different kind of binding surface than normal cell wall. If misalignment of microfibrils within dislocations is due to fiber bending, the microfibrils may be both compressed and stretched depending on their location within the cell, and it is not unthinkable that contracted or elongated glycosidic bonds may exist in some positions. An otherwise recognisable binding surface to a CBM may thus turn into a non-recognisable one, while making it recognisable to another CBM. It has been shown that minute differences in cellulose structure may affect the binding affinity of closely related CBMs differently (Boraston et al. 2003).

While it could be that the R values calculated for CBHI, CBHII and EGII are slightly greater than 1 (R ~ 1.1–1.2, Fig. 8b), i.e. that these enzymes actually do have a slight preference for dislocations, their standard deviations (not shown, not standard error of the means as shown in Fig. 8b) does not allow us to make a stricter interpretation towards this end. For CBHI and CBHII the value of R ~1 is in line with the finding of Thygesen et al. (2007) that microfibrils continue through dislocations. This continuity would mean that there is no increase in the number of free cellulose chain ends within dislocations and consequently the activity of exocellulases would not be expected to be any higher than within the surrounding cell wall. It is however appropriate to remark here that in a number of microscopical observations using fluorescently labelled enzymes (summarized in Hidayat et al. 2012) increased fluorescence has been observed in areas resembling or which can be interpreted as dislocations. None of these, however, investigated *H. insolens* EGV. Filonova et al. (2007) showed that CBM1. *T. reesei* Cel7A distinctly binds to what seems to be dislocations in spruce pulp fibers. As in their experiment standard fluorescence microscopy (instead of CLSM) was used, no information on the penetration of the enzyme into the fibers could be extracted. The binding was described to occur in the S1 layer of the fiber which was stated to be intact post-treatment. In our study the procedure chosen to select SOIs may have meant that (almost) no S1 was included. This might perhaps partly explain the different results obtained. Other non-CLSM fluorescence microscopy studies showing enzymes binding to dislocations involved CBMs that were not used in our study (CBM3 and CBM28 of *Clostridium josui* on Japan cedar wood (Kawakubo et al. 2010), CBM6 of *Clostridium thermocellum* on *Valonia ventricusa* cellulose (Ding et al. 2006), CBM1 from *Phanerochaete chrysoporum* Cel7D on delignified spruce/birch pulp fibers [Hildén et al. 2003]). Recently, swollenin has also been shown, by use of CLSM combined with other techniques, to target and disrupt dislocations (Gourlay et al. 2015). In contrast to these results Arantes et al. (2014) showed that enzymatic hydrolysis of fibers takes place primarily by peeling from the surface, not by segmentation.

In a number of hydrolysis experiments with enzyme preparations that do not contain *H. insolens* EGV, fibers have nevertheless been observed to break at dislocations (summarized in Hidayat et al. 2012). If it is true that for most cellulases no increased enzyme binding occurs within dislocations, then perhaps the segmentation phenomenon is better understood from a mechanical viewpoint. That is: dislocations are mechanically weak locations, which function as crack initiation points when the fibers are subjected to mechanical agitation (Baley 2004; Forgacs 1961; Page et al. 1972). In a recent study we showed that the development in fiber lengths during hydrolysis may indeed be successfully modelled based purely on the simple mechanical principles of 3-point bending (Thygesen et al. 2014). Thus our findings do not support the notion that the mechanisms involved in particle size reduction generally depend upon preferred binding of cellulases to dislocations.

In conclusion, in this work a CLSM-based method has been developed to measure the abundance of an enzyme within dislocations relative to the surrounding cell wall. The method assesses mainly the S2 layer of the cell wall and is shown to be able to discern the preferred binding to dislocations of *Humicola insolens* EGV from the rest of the enzymes tested. Since cellulases for which segmentation of fibers at dislocations have been observed in other studies did not show a significant difference in binding between dislocations and the surrounding cell wall, the present study do not support the idea that preferred binding of cellulases to dislocations is generally important for the observed segmentation at dislocations during liquefaction.
